# Cross-sectional survey of attitudes and beliefs towards dementia risk reduction among Australian older adults

**DOI:** 10.1186/s12889-023-15843-0

**Published:** 2023-05-30

**Authors:** Joyce Siette, Laura Dodds, Kay Deckers, Sebastian Köhler, Christopher J. Armitage

**Affiliations:** 1 MARCS Institute for Brain, Behaviour and Development, Western Sydney University, Sydney, NSW 2145 Australia; 2 Centre for Health Systems and Safety Research, Australian Institute of Health Innovation, Macquarie University, Macquarie Park, NSW 2109 Australia; 3 Alzheimer Centrum Limburg, Department of Psychiatry and Neuropsychology, School for Mental Health and Neuroscience, Maastricht University, Maastricht, 6200 MD the Netherlands; 4 Manchester Centre for Health Psychology, University of Manchester, Manchester, M13 9PL UK; 5 Manchester University NHS Foundation Trust, Manchester Academic Health Science Centre, Manchester, M13 9PL UK; 6 NIHR Greater Manchester Patient Safety Translational Research Centre, University of Manchester, Manchester, M13 9PL UK

**Keywords:** Dementia risk, Motivation, Older adults, Attitudes

## Abstract

**Background:**

Little is known about what drives older adults’ motivation to change their behaviour and whether that is associated with their personal dementia risk profile. Our aims were to (i) understand what sociodemographic factors are associated with older Australians’ motivation to change behaviour to reduce their dementia risk, and (ii) explore the relationship between socio-demographic factors and motivation to reduce dementia risk with health- and lifestyle-based dementia risk scores in older adults.

**Methods:**

A cross-sectional online postal or telephone survey was administered to community-dwelling older adults in New South Wales, Australia between January and March 2021. Measures included socioeconomic status, locality, and health status, the Motivation to Change Lifestyle and Health Behaviours for Dementia Risk Reduction (MCLHB-DRR) scale and the lifestyle-based dementia risk score (LIBRA index). Multiple linear regression analyses were used to explore the associations for (i) sociodemographic factors and motivation to reduce dementia risk (MCLHB-DRR scales) and (ii) sociodemographic factors and motivation to reduce dementia risk with health- and lifestyle-based dementia risk (LIBRA index).

**Results:**

A total of 857 older adults (mean age 73.3 years, SD = 6.0, range 65–94; 70% women; 34.6% less than grade 6 education) completed the survey. Respondents reported high levels of motivation to adopt behaviour changes, agreeing on the importance of good health. Individuals who were younger were more likely to have greater motivation to modify lifestyle to reduce dementia risk and had higher perceived benefits to gain by adopting a healthy lifestyle. Dementia risk scores were moderately low (mean LIBRA index =− 2.8 [SD = 2.0], range − 5.9–3.8), indicating relatively moderate-to-good brain health. Men with low socioeconomic status and higher perceived barriers to lifestyle change had higher dementia risk scores.

**Conclusions:**

Public health campaigns need to overcome motivational barriers to support reductions in dementia risk. A multifaceted and inclusive approach targeting both sociodemographic differences and impediments to brain healthy lifestyles is required to achieve genuine change.

**Trial registration:**

ACTRN12621000165886, Date of registration: 17/02/2021.

**Supplementary Information:**

The online version contains supplementary material available at 10.1186/s12889-023-15843-0.

## Introduction

Dementia is a neurodegenerative disorder with profound psychological, physical, social and economic implications for diagnosed individuals, their families and more broadly, society [1, 2]. Internationally, approximately 50 million people are affected by dementia, with this figure projected to triple by 2050 [3]. Despite extensive multinational efforts, there are currently no widely-available disease-modifying therapies for dementia [4]. However, increasing bodies of evidence have emphasized the role of modifiable risk factors that can exacerbate or delay the onset of dementia in mid- and late-life [5]. A focus on employing strategies targeting policies, legislation and, population-wide programs to reduce risk, diagnose and care for individuals with dementia have recently been implemented [1].

Research highlights the contribution of multiple modifiable risk factors in increasing the risk of late-life dementia development, with recent estimates indicating that these factors potentially account for a substantial part of dementia cases globally [6], with emerging additional factors [6–8]. These factors include physical inactivity, presence of depression, diabetes, hypertension, smoking, obesity, coronary heart disease, chronic kidney diseases, hypercholesterolemia, midlife hearing loss, low mental and cognitive activity, excessive alcohol consumption, poor diet, and social isolation [6–8]. Targeting these modifiable risk factors by encouraging people to engage in healthy living behaviors have shown promise as risk reduction strategies and provide avenues for further research [1, 9–12]. However, health behaviour change is challenging and multifaceted [13].

Understanding the barriers towards adopting brain-healthy behaviours is required to tailor effective interventions targeting dementia prevention. In order to do so, a number of health behaviour models (e.g., Health Belief Model, HBM [14, 15]) have been developed to understand the processes that underlie individual behaviour [14, 15]. Major determinants of health behaviour change include awareness and knowledge of the disease and its modifiable risk factors, perceived attitudes (such as susceptibility and severity of the disease), motivation (such as perceived benefits or barriers for completing risk-reducing behaviours), and other influences (such as social and physical environment), which impact the ultimate step to action [14, 16–19].

To date, there are only a handful of large-scale quantitative studies exploring motivation towards dementia risk reduction behaviours in the general adult population, including health beliefs, awareness and attitudes [19–23]. A study of Dutch adults observed high scores for general health motivation and perceived benefits, and low scores on perceived susceptibility, severity, barriers, cues to action and self-efficacy. Older participants found dementia to be a more severe disease and reported fewer benefits and barriers of performing health-enhancing behaviour compared to younger participants [21]. Similarly, a few studies conducted in Australia, United States and Turkey found that younger age was positively associated with the intention to adopt a healthy lifestyle [20, 22, 23]. They also reported that having better perceived benefits and lower barriers, better self-efficacy and more knowledge about dementia risk reduction was linked with the intention to adopt a healthy lifestyle. Akyol et al. (2020) further described that men and individuals with low levels of education and income had higher perceived barriers and lower general health motivation [20].

However, the above-described studies examining motivation to change behaviour are hampered by several limitations. Firstly, the low participation rates (e.g., 17%, [21]) and highly educated participants (e.g., [21]) limit generalizability to the general population. Indeed, most studies exploring motivational levels surveyed young or middle-aged adults [19, 23], those residing in metropolitan areas and individuals from a higher socioeconomic status [19–23]. To our knowledge, none of the previous studies investigated whether motivators for adopting and maintaining a healthier lifestyle for dementia risk reduction are dependent on an individual’s health- and lifestyle-based susceptibility to develop dementia.

Therefore, the aims of this study were to (i) examine socio-demographic factors associated with motivation to reduce dementia risk among older Australians; and (ii) identify the relationship between socio-demographic factors, motivation towards dementia risk reduction with higher health- and lifestyle-based dementia risk scores in older adults.

## Methods

### Study design

This was a cross-sectional online, postal or telephone survey study, which was part of the BRAIN BOOTCAMP^TM^ research program targeting reduced dementia risk and increased dementia literacy for older adults. This was an open survey and tested for functionality by contributing investigators before fielding the questionnaire. The present study describes baseline assessment of motivation towards dementia risk reduction as well as personal dementia risk profiles pre-entry to the program. The primary outcomes for this study were motivation towards dementia risk reduction behaviours and dementia risk. The design of BRAIN BOOTCAMP^TM^ has been described in detail within Siette et al. 2022 [24].

### Study population and recruitment

The target population was community-dwelling adults aged over 65 years old residing in New South Wales (NSW), Australia. Individuals were excluded if they had a self-reported active episode of major depression, and/or an existing diagnosis of dementia, and/or are currently enrolled in any lifestyle change intervention. Participants were also excluded if they only partially completed the survey. Ethical approval for the study was given by the Macquarie University Human Ethics Committee (ethics reference number 9174). All participants were provided with a participant information and consent form, advising of the purpose of the study, who the main investigators were, the approximate length of the survey, and that their data would be de-identified and stored securely on password protected servers, only accessed by university approved researchers. Once participants viewed the form, they provided informed consent and all methods were performed in accordance with the Declaration of Helsinki. Sample size was estimated based on a significance level of alpha (5%, two-sided) and 80% power, according to proof-of-concept trials and methodologies used in previous studies on similar topics to detect group differences [24], requiring a sample of 176 participants.

A convenience sampling approach was used to recruit participants through a broad advertisement scheme through offline and online media as well as banners. Details about the study were circulated throughout New South Wales in various forms of media (i.e., newsletters, flyers, radio), through large organisations (e.g., Council of the Ageing NSW, Health Consumers NSW), and through e-newsletters and flyers at local councils to facilitate the inclusion of individuals from low socioeconomic backgrounds and geographically-diverse areas. Further information about recruitment efforts and survey announcements are detailed in Siette et al. 2022 [24].

Individuals were eligible to participate in the study if they met all the following criteria: (i) older than 65 years of age, (ii) no confirmed or self-diagnosis of dementia, and (iii) provided informed consent. The survey was distributed over seven weeks between 18 January to 10 March 2021.

### Procedure

Individuals who wished to be involved in the study were directed to the BRAIN BOOTCAMP^TM^ website (http://www.brainbootcamp.com.au), where they completed an online survey via a separate Qualtrics link that assessed their motivation to change their behaviour. The online survey was 26 pages with an average of 4 items per page. Participants could track their progress with a progress bar at the top of the survey. Completeness checks were used before proceeding to the next page of the survey flagging mandatory questions, and participants were able to go back to review their answers before the final submission of their survey. Participants who could not complete the online survey (e.g., due to a lack of internet access) could post their answers through a mailed-out version of the survey or were interviewed over the phone. Interviewers followed the script of the online survey to elicit participant responses.

### Measurements

#### Demographics

Sociodemographic variables included age, gender and education, where education was divided into three levels: high (> 12 years, i.e., higher vocational education or university), middle (between 7 and 12 years, i.e., intermediate secondary education or intermediate vocational education or university) and low (between 0 and 6 years, e.g., primary or low vocational education). The Accessibility/Remoteness Index of Australia (ARIA) [25] was used to measure participants remoteness, and divides Australia into five classes of remoteness on the basis of a measure of relative access to services (major city, inner regional, outer regional, remote, very remote).

The Socio-Economic Indexes for Areas (SEIFA) is an Australian Bureau of Statistics product that ranks areas in Australia according to relative socioeconomic advantage and disadvantage based on information from the five-yearly Census of Population and Housing. To calculate socioeconomic status, the Index of Relative Socioeconomic Advantage and Disadvantage (IRSAD) [26] was used, which measures income, education, employment type, presence of disability and unemployment, proportion of single parent families with dependents and proportion of low rent housing.

Health status was assessed through a series of yes/no responses to different health conditions (e.g., heart disease, diabetes, hypertension, high cholesterol) with obesity determined by self-reported weight and height to calculate body mass index (kg/m^2^).

#### Motivation towards dementia risk reduction behaviours

The Motivation to Change Lifestyle and Health Behaviours for Dementia Risk Reduction (MCLHB-DRR) scale was used to measure participants’ attitudes and beliefs towards modifying their behaviours [19]. Specific to dementia risk reduction and developed for the Australian sample, this 27-item scale was built upon the assumptions of the Health Belief Model (HBM) [27]. The MCLHB-DRR scale has demonstrated high to moderate internal reliability (α = 0.61–0.86) and test-retest reliability (α = 0.55–0.78) and is typically described across its 7 subscales. These 7 subscales each measure different perceptions of the disease including (i) consequences of the condition, (ii) the risk of getting the condition, (iii) perceived barriers, and (iv) benefits of engaging in the health-promoting behaviour, (v) general health motivation, (vi) self-efficacy, and (vii) external stimuli that may trigger an individual to take action [19]. Examples of questions from each of the subscales are provided in Supplementary Material. These items were answered on a 5-point Likert-scale, ranging from “strongly disagree” [1] to “strongly agree” [5]. Item scores were summed up to yield subscale scores, with higher scores indicating higher motivation to change lifestyle and behaviour.

#### Dementia risk score

Dementia risk was measured using the LIfestyle for BRAin health (LIBRA) index, which was developed as an instrument based on a systematic review and Delphi consensus study and has been associated with cognitive functioning/decline and dementia risk in midlife and late life [28–34]. The index consists of a weighted sum score of 12 modifiable risk and protective factors including medical history, lifestyle factors (smoking and alcohol consumption), depressive symptoms assessed via Patient-Health Questionnaire (PHQ-9) [35], cognitive activity through Cognitive Reserve Questionnaire (CRIq) [36], physical activity, and diet adherence (Mediterranean Diet Adherence Screener (MEDAS) [37] to have a weighted sum score ranging from − 5.9 to + 12.7 where higher scores indicated greater risk of developing dementia [28].

### Statistical analysis

Participant characteristics including sociodemographic variables, MCLHB-DRR subscale scores, and the LIBRA index were described using descriptive statistics. Data were analysed on a complete case with missing data omitted. Outliers were identified using casewise diagnostics and removed from analysis. Differences between certain demographic subgroups on the MCLHB-DRR subscale scores were analysed using independent t-tests (normally distributed continuous variables for two groups), one-way ANOVA (normally distributed continuous variables for three groups) and Chi-squared (categorical) tests for the different types of variables. Two separate linear regression models were applied to address the study aims. The primary outcome variables (average responses on a Likert scale) were considered continuous in nature. The first regression analysis was used to evaluate the association between demographic variables on separate MCLHB-DRR subscale scores. The second regression evaluated predictors of high LIBRA index scores including sociodemographic, health conditions, and individual MCLHB-DRR subscale scores. Selection of factors used in the models for both primary outcomes (i.e., motivation towards dementia risk reduction [MCLHB-DRR subscales], dementia risk [LIBRA index]) were guided by previous literature using the physical-environmental model [38, 39] and based on prior knowledge of potential associations. Separate regression analyses, using step-wise entry, were used to examine predictors of both primary outcomes. For all models, the first step involved entering all predictors in one basic model according to their effect sizes. In step two, predictors were entered one by one in order of their standardized beta coefficients (ßs) in the previous analysis as long as they contributed to the model. Regression diagnostics were used to identify assumptions of normality, linearity, multicollinearity and homoscedasticity (e.g., intercorrelations, tolerances and variance inflation factors (VIF) exploration). The models were also examined by visual inspection of the distributions and normal probability plots of their standardized residuals. Statistical significance was inferred at p ≤ 0.05 in two-sided tests. All analyses were conducted using SPSS Version 27 for Windows [40].

## Results

### Sample characteristics

Sample characteristics are summarized in Table 1. A total of 880 individuals responded to the survey (856 participants completed all questions; 10 postal, 1 phone, 845 online). Average time to complete the survey was 38.3 min (SD = 21.4; range = 10.6-145.8). The sample’s mean age was 73.3 years (SD = 6.0, range = 65–94), mostly women (70.0%) and living in a major city (77.7%). Most had completed both primary and intermediate secondary education (55.4%), with nearly half of the sample having attained graduate studies (44.6%). Most respondents were born in an English-speaking country (84.1%) and nearly half of the sample (49.4%) were from a high socioeconomic background. Respondents self-reported having a multitude of chronic health conditions, with over three quarters reporting at least one long-term health condition. The most common condition was self-reported high cholesterol (51.9%), followed by high blood pressure (47.5%) and heart disease (24.9%). Compared to available national census data [41, 42], the sample had similar proportions for heart disease, education attainment and locality but came from a higher socioeconomic background compared to the national average (49.4% vs. 34%).

**Table 1:** Summary of study participant demographics (N = 857)

Characteristic	N (%)
**Gender**	
Women	597 (70.0)
Men	256 (30.0)
**Age (Mean [SD], range)**	73.4 [6.1], 65–94
65–69	276 (32.2)
70–79	444 (51.8)
80–89	121 (14.1)
90+	15 (1.8)
**Education**	
Low	293 (34.6)
Middle	176 (20.8)
High	377 (44.6)
**Country of birth**	
English-speaking country	719 (84.1)
Non-English speaking country	136 (15.9)
**Locality**	
Major city	627 (77.7)
Regional/Remote	176 (22.3)
**Socioeconomic status**	
1 (lowest)	43 (5.4)
2	125 (15.8)
3	138 (17.4)
4	95 (12.0)
5 (highest)	391 (49.4)
**Health status (N, %)** ^**1**^	
Heart condition	211 (24.9)
Kidney disease	29 (3.4)
Diabetes	77 (9.0)
High cholesterol	445 (51.9)
High blood pressure	407 (47.5)
Smoking	17 (2.0)
Obesity	203 (24.2)
Depression	16 (1.9)

^*1*^ *Number of participants who responded with ‘Yes’. NB: Numbers to not add to 857 due to some missing data for some variables.*

### Levels of motivation and association with demographics and health status

**Fig. 1:**
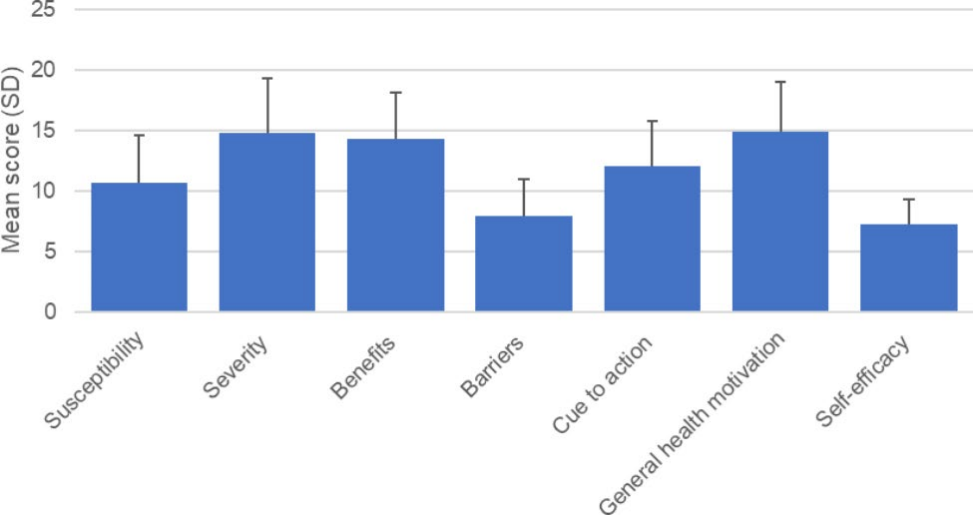
Average score by sub-scale item on the Motivation towards dementia risk reduction (MCLHB-DRR) scale

Overall, all subscale scores were approximately normally distributed. Mean individual item response scores of the MCLHB-DRR subscales are summarized in Fig. 1 and ranged from 2.0 (SD = 0.8; item 14 identified barrier as “too busy”) to 4.2 (SD = 0.8; item 22 “nothing is as important as good health”).

For five MCLHB-DRR scales, respondents had higher agreement values. Older adults had high agreement that they would feel anxious and stressed if they developed dementia (perceived severity mean score 14.8 [3.9], maximum score 20), believed that changing lifestyle and health habits would help to prevent chances of developing dementia (perceived benefits mean score 14.3 [3.8], maximum score 20), and valued their general health and wellbeing (general health motivation mean score 14.9 [4.1], maximum score 20). Respondents also had high levels of agreement in being able to confidently change lifestyle and health behaviour for dementia risk reduction (self-efficacy mean score 7.2 [2.1], maximum score 10), and strongly agreed that having social influences would change their lifestyle and health behaviour for dementia risk reduction (cues to action mean score 12.0 [3.8], maximum score 20).

Respondents had low agreement for two MCLHB-DRR scales: Reporting on possible barriers associated with changing lifestyle and health behaviour to reduce dementia risk were low (perceived barriers mean score 8.3 [2.6], maximum score 20). Participants also had low agreement on risk for developing dementia during their lifetime (perceived susceptibility mean score 11.1 [3.3], maximum score 25).

Univariate associations are reported in Supplementary Table S1. Younger age was significantly associated with five subscales, perceived higher severity, benefits, cues to action, general health motivation and self-efficacy. Additional associations from low socioeconomic status, locality and some health conditions were found with perceived severity and barriers and general health motivation. Multiple linear models for six of the MCLHB-DRR subscales were significant (Tables 2 and 3).

**Table 2:** Predictors of motivators to change lifestyle and healthy behaviours in older adults across three subscales (perceived susceptibility, perceived severity, perceived benefits).

	Perceived susceptibility			Perceived severity			Perceived benefits		
	ß	CI (95%)	p-value	ß	CI (95%)	p-value	ß	CI (95%)	p-value
**Gender**									
Women	-0.45	-0.97-0.06	0.09	**0.93**	**0.38–1.42**	**< 0.001**	0.20	-0.19-0.59	0.31
Men	1			1			1		
**Age**									
65–69	**1.12**	**0.36–1.88**	**0.004**	1.44	**0.63–2.24**	**< 0.001**	**1.65**	**1.07–2.23**	**< 0.001**
70–79	0.32	-0.37-1.02	0.362	0.53	-0.20-1.27	0.16	**1.36**	**0.83–1.89**	**< 0.001**
80+	1			1			1		
**Country of birth**									
English-speaking country	0.37	-0.28-1.02	0.27	-0.46	-1.15-0.23	0.19	-0.47	-0.96-0.02	0.06
Non-English speaking country	1			1			1		
**Locality**									
Major city	-0.37	-1.09-0.35	0.31	0.70	-0.06-1.46	0.07	-0.02	-0.57-0.52	0.94
Regional/remote	1			1			1		
**Socioeconomic status**									
1 (lowest)	-0.001	-1.24-1.24	0.99	0.72	-0.59-2.03	0.28	0.47	-0.47-1.41	0.33
2	-0.46	-1.27-0.35	0.26	0.15	-0.70-1.01	0.73	-0.06	-0.67-0.56	0.86
3	0.10	-0.58-0.78	0.77	0.39	-0.36-0.11	0.29	-0.15	-0.67-0.37	0.56
4	0.09	-0.66-0.84	0.81	0.39	-0.10-1.48	0.09	**0.70**	**0.13–1.26**	**0.02**
5 (highest)	1			1			1		
**Education**									
Low	**0.69**	**0.14–1.23**	**0.01**	**0.58**	**0.001–1.15**	**0.05**	0.16	-0.25-0.58	0.43
Medium	0.40	-0.20-1.01	0.19	-0.24	-0.88-0.40	0.46	-0.22	-0.68-0.24	0.34
High	1			1			1		
**Heart disease**	-0.33	-0.89-0.23	0.24	-0.05	-0.64-0.54	0.88	-0.25	-0.68-0.17	0.25
**Kidney disease**	0.32	-0.91-1.55	0.61	-0.16	-1.46-1.15	0.82	-0.01	-0.94-0.93	0.99
**Diabetes**	-1.12	-0.76-0.53	0.72	-0.06	-0.74-0.62	0.87	0.11	-0.38-0.60	0.66
**Cholesterol**	**0.42**	**0.08–0.76**	**0.02**	0.04	-0.32-0.40	0.82	0.09	-0.17-0.35	0.49
**Smoking**	1.06	-0.11-2.22	0.08	1.11	-0.13-2.34	0.08	-0.27	-1.15-0.62	0.55
**Obesity**	0.23	-0.12-0.57	0.20	0.14	-0.23-0.50	0.46	**0.33**	**0.07–0.59**	**0.01**
**High blood pressure**	0.10	-0.21-0.40	0.53	0.27	-0.05-0.59	0.10	0.09	-0.14-0.32	0.43
**Alcohol**	-0.29	-0.87-0.29	0.33	-0.09	-0.69-0.52	0.79	**-0.59**	**-1.03—0.15**	**0.008**
**Depression**	0.45	-0.34-1.24	0.27	-0.03	-0.87-0.81	0.94	-0.19	-0.79-0.41	0.53
	F(20,743) = 2.71		0.004	F(20,743) = 11.98		< 0.001	F(20,743) = 6.18		< 0.001

β = standardized beta; CI = confidence interval

**Table 3:** **Predictors of motivators to change lifestyle and healthy behaviours in older adults across four subscales (barriers, cues to action, general health motivation, self-efficacy)**

	Barriers			Cues to action			General health motivation			Self-efficacy			
	ß	CI (95%)	p-value	ß	CI (95%)	p-value	ß	CI (95%)	p-value	ß	CI (95%)	p-value	
**Gender**													
Women	**0.44**	**0.03–0.85**	**0.04**	**0.49**	**0.05–0.93**	**0.03**	0.17	-0.22-0.55	0.40	0.08	-0.14-30	0.48	
Men	1			1			1			1			
**Age**													
65–69	-0.05	-0.65-0.56	0.89	**1.27**	**0.62–1.92**	**< 0.001**	**1.19**	**0.62–1.78**	**< 0.001**	**0.59**	**0.27–0.92**	**< 0.001**	
70–79	-0.41	-0.96-0.15	0.15	**0.77**	**0.17–1.36**	**0.01**	**0.87**	**0.35–1.39**	**< 0.001**	**0.54**	**0.24–0.83**	**< 0.001**	
80+	1			1			1			1			
**Country of birth**													
English-speaking country	-0.30	-0.82-0.22	0.25	-0.22	-0.78-0.34	0.44	**-0.76**	**-1.24—0.27**	**0.002**	-0.19	-0.47-0.09	0.18	
Non-English speaking country	1			1			1			1			
**Locality**													
Major city	0.14	-0.43-0.71	0.633	0.06	-0.55-0.67	0.85	0.13	-0.41-0.66	0.64	-0.06	-0.37-0.25	0.39	
Regional/remote	1			1			1			1			
**Socioeconomic status**													
1 (lowest)	**1.05**	**0.06–2.03**	**0.04**	0.26	-0.87-1.32	0.63	-0.25	-1.17-0.68	0.60	-0.16	-0.69-0.37	0.56	
2	0.14	-0.50-0.78	0.66	-0.18	-0.87-0.52	0.62	0.20	-0.41-0.80	0.53	-0.02	-0.36-0.33	0.93	
3	0.36	-0.19-0.90	0.20	-0.35	-0.94-0.23	0.24	-0.22	-0.73-0.29	0.39	-0.25	-0.54-0.04	0.09	
4	0.27	-0.32-0.87	0.37	0.23	-0.41-0.86	0.49	0.42	-0.14-0.98	0.14	0.22	-0.10-0.54	0.17	
5 (highest)	1			1			1			1			
**Education**													
Low	**0.52**	**0.09–0.95**	**0.02**	**0.76**	**0.30–1.22**	**0.001**	0.40	-0.004-0.81	0.05	0.06	-0.17-0.29	0.60	
Medium	**0.50**	**0.02–0.99**	**0.04**	**0.70**	**0.18–1.22**	**0.008**	0.15	-0.30-0.61	0.50	0.16	-0.10-0.42	0.23	
High	1			1			1			1			
**Heart disease**	-0.13	-0.57-0.32	0.58	-0.29	-0.76-0.19	0.24	-0.24	-0.66-0.18	0.26	0.03	-0.21-0.26	0.84	
**Kidney disease**	0.31	-0.67-1.29	0.53	-0.31	-1.36-0.74	0.56	-0.47	-1.39-0.45	0.32	-0.05	-0.58-0.48	0.86	
**Diabetes**	0.08	-0.44-0.59	0.78	0.19	-0.36-0.74	0.49	0.39	-0.09-0.87	0.11	0.10	-0.18-0.38	0.48	
**Cholesterol**	-0.06	-0.33-0.22	0.69	0.23	-0.06-0.52	0.13	-0.01	-0.25-0.25	0.99	0.08	-0.07-0.22	0.31	
**Smoking**	**1.08**	**0.15–2.01**	**0.02**	0.46	-0.53-1.46	0.36	-0.17	-1.04-0.71	0.71	-0.06	-0.56-0.45	0.83	
**Obesity**	**0.30**	**0.02–0.57**	**0.04**	0.22	-0.07-0.52	0.14	0.02	-0.24-0.28	0.88	-0.08	-0.23-0.07	0.29	
**High blood pressure**	0.22	-0.02-0.46	0.07	0.05	-0.21-0.36	0.73	-0.04	-0.27-0.18	0.72	0.003	-0.13-0.13	0.97	
**Alcohol**	-0.12	-0.58-0.34	0.62	**-0.89**	**-1.38—0.39**	**< 0.001**	-0.06	-0.49-0.67	0.78	**-0.29**	**-0.54—0.05**	**0.02**	
**Depression**	**0.78**	**0.15–1.41**	**0.02**	0.05	-0.67-0.68	0.99	0.45	-0.14-1.04	0.14	0.28	-0.06-0.62	0.11	
	F(20,743) = 6.80		< 0.001	F(20,7-0.8943) = 7.81		< 0.001	F(20,743) = 5.96		< 0.001	F(20,743) = 1.97		0.02	

β = standardized beta; CI = confidence interval

Models found that men had higher perceived risk of developing dementia compared to women (p = 0.03). Regression analysis also found that compared to adults > 80 + years, adults younger than 80 years had higher perceived anxiety on dementia development (ß = 1.12, p < 0.001), stronger beliefs that changing lifestyle and health habits would assist in reducing dementia risk (ß = 1.65, p < 0.001), higher general health motivation (ß = 1.19, p < 0.001), greater confidence in their ability to change lifestyle and health behaviour (ß = 1.27, p < 0.001), and stronger beliefs that having social influences would change their lifestyle and health behaviour for dementia risk reduction (ß = 0.59, p < 0.001).

Respondents with low levels of education reported stronger beliefs in having social influences to influence their lifestyle as compared to individuals who had high levels of education (ß = 0.76, p = 0.001). They also had higher perceived barriers (ß = 0.52, p = 0.02), perceived susceptibility (ß = 0.69, p = 0.01), and severity (ß = 0.58, p = 0.05).

In terms of health conditions, older adults who had high cholesterol had more perceived susceptibility to developing dementia (ß = 0.42, p = 0.02) when compared to adults who did not have self-reported high cholesterol. Older adults who identified as heavy alcohol drinkers reported fewer benefits to changing lifestyle to reduce risk (ß = -0.59, p < 0.01), had fewer perceived social influences (ß = -0.89, p < 0.001) to modify their behaviour towards dementia risk reduction, and lower self-efficacy (ß = -0.29, p = 0.02). Older adults who identified as being obese had higher perceived benefits (ß = 0.33, p = 0.03) and barriers (ß = 1.08, p = 0.02).

### Effect of sociodemographic and motivational attitudes on LIBRA Index

**Fig. 2:**
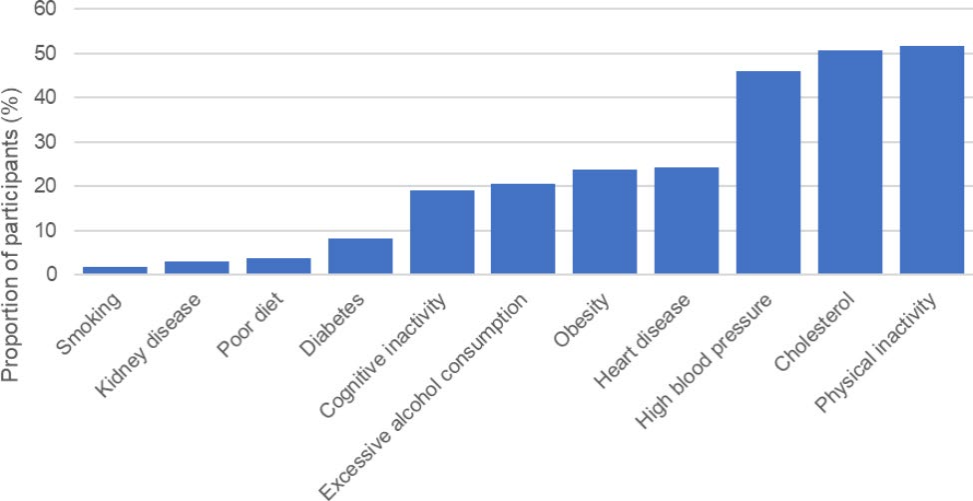
Proportion of participants with LIBRA index risk factors

Respondents had good to moderate brain health scores with a mean LIBRA index of -2.8 (SD = 2.0, range − 5.9–3.8) with 8.8% obtaining the highest possible score (-5.9). The proportion of respondents and the presence of each risk factor is summarized in Fig. 2 and Supplementary Table S2. Univariate analysis found that women had lower risk (p = 0.04) and individuals from a low socioeconomic status had higher dementia risk (p < 0.001) (Supplementary Table S3). After accounting for other factors in the final linear regression model, women (ß = -0.35, p = 0.02), younger participants (ß = -0.72, p = 0.001), participants who were from a higher socioeconomic status (ß = 0.99, p < 0.01), and those reporting less perceived susceptibility (ß = 0.05, p = 0.04), more benefits (ß = 0.07, p = 0.03) and fewer barriers (ß = 0.10, p < 0.05) to adopting a healthy lifestyle had significantly lower dementia risk scores (R^2^ = 0.97, F(18,745) = 3.56, p < 0.001) (Table 4).

**Table 4:** Predictors of health- and lifestyle-based dementia risk scores (LIBRA index) in older adults

	LIBRA index			
	Mean (SD)	ß	CI (95%)	p-value
**Gender**				
Women	-2.9 (1.9)	**-0.35**	**-0.64—0.52**	**0.02**
Men	-2.6 (2.0)	1		
**Age**				
65–69	-2.9 (1.8)	**-0.72**	**-1.16—0.29**	**0.001**
70–79	-2.8 (2.0)	-0.36	-0.76-0.04	0.08
80+	-2.5 (2.1)	1		
**Country of birth**				
English-speaking country	-2.8 (1.9)	0.23	-0.15-0.61	0.23
Non-English speaking country	-2.9 (2.1)	1		
**Locality**				
Major city	-2.9 (2.0)	0.20	-0.22-0.61	0.35
Regional/remote	-2.5 (1.8)	1		
**Socioeconomic status**				
1 (lowest)	-1.9 (2.2)	**0.99**	**0.28–1.70**	**0.01**
2	-2.6 (2.0)	**0.66**	**0.19–1.12**	**0.01**
3	-2.5 (1.9)	**0.54**	**0.15–0.93**	**0.01**
4	-2.9 (1.9)	0.04	-0.40-0.47	0.87
5 (highest)	-3.0 (1.9)	1		
**Education**				
Low	-2.8 (2.0)	-0.20	-0.51-0.12	0.22
Medium	-2.6 (1.9)	0.15	-0.20-0.50	0.41
High	-2.9 (1.9)	1		
**Perceived susceptibility**	**-**	**0.05**	**0.003-0.10**	**0.04**
**Perceived severity**	-	0.01	-0.04-0.05	0.82
**Perceived benefits**	**-**	**0.07**	**0.01–0.14**	**0.03**
**Barriers**	**-**	**0.10**	**0.05–0.15**	**< 0.001**
**Cues to action**	-	0.01	-0.05-0.07	0.77
**General health motivation**	-	0.01	-0.06-0.07	0.83
**Self-efficacy**	-	-0.04	-0.16-0.08	0.52
		F(18,745) = 3.56		< 0.001

β = standardized beta; CI = confidence interval

## Discussion

The relationship between dementia and modifiable lifestyle behaviours has prompted action for a public health approach, incorporating population-based risk-reduction strategies, to tackle dementia prevention. The present study is one of the first to investigate older Australians’ motivation and actions towards brain health and dementia risk reduction. The findings reveal group differences in readiness to take action and indicate a role for continued efforts to increase awareness and motivation in older age, individuals with low socioeconomic status, as well as reducing barriers to changing behaviour and lifestyle.

### Motivation towards dementia risk reduction behaviours

Most of our findings on motivation towards behaviours and habits surrounding dementia risk reduction align with previous literature published with Australian populations [19, 23, 43]. Our sample was highly motivated to adopt and adapt lifestyle changes, with a large proportion of older adults agreeing on the importance of good health and paying attention to their health [19]. In general, motivation to change behaviour based on severity, self-efficacy, cues to action and perceived susceptibility to dementia were higher compared to 30–80-year-old Dutch [44] and > 40 year-old Turkish general populations [20]. Despite our older adult sample, participants reported higher self-efficacy to change behaviour to reduce dementia risk. Findings varied for domains of perceived benefits and general health motivation, which were higher compared to Dutch (2021) [21], but lower compared to Turkish populations [20]. However, comparisons are limited due to the different sample ages, cultural backgrounds and sample sizes.

Age-associated differences on motivation towards dementia risk reduction behaviours were apparent. Similar to earlier, international work in Dutch adults [21], participants aged younger than 79 years perceived dementia as a more severe disease, expressed that modifying health behaviour had fewer benefits, more barriers and reported less confidence to perform the health-related behaviour. This may be because older age is associated with better emotion regulation capacity [45], psychological and emotional resilience [45] and frequent pro-hedonic selection and motivation compared to younger cohorts [46], which can result in greater positive health outcomes [45]. These findings have persisted throughout COVID-19 [47, 48], the same period in which our study was launched, thus a possible explanation for why younger participants were less motivated. We found that barriers to lifestyle change persist amongst older adults, and of these, lack of knowledge regarding potential for dementia risk reduction may be the most common [23, 43]. Indeed, newly developed attitudes are formed on the basis of information, and older adults may be known to lack confidence when faced with minimal information and may avoid making decisions [49].

Our findings further highlight the complex systemic and structural forces that have a disproportionate impact on those in lower socioeconomic areas. It is well established that socioeconomic status, income and education are inextricably linked and that a health gradient exists wherein those with lower income, educational attainment and occupational status have higher risks of nearly every cause of premature morbidity and mortality [50, 51]. Thus, socioeconomic status has a strong influence on the lack of motivation to adopt healthier behaviours. Addressing the social determinants of health (e.g., early education, urban planning, community development, employment) as well as adopting community level engagement strategies and interventions targeted at lower socioeconomic individuals have improved health outcomes by influencing attitudes, increasing public knowledge and creating opportunities for healthy choices [38]. Our study demonstrates that this is also pertinent in the context of modifiable dementia risk and societal and public health efforts should emphasise the importance of making information and resources available, accessible and relatable for lower socioeconomic areas [29].

Beliefs to adopt healthier lifestyles and behaviour varied somewhat between genders, with men more likely to perceive themselves as ‘at risk’ for developing dementia in the future. Unlike prior research, we did not find a gender difference for self-efficacy, which suggests that men were more confident in the possibility of reducing dementia risk compared to women [52], possibly due to gender-specific beliefs and the rooted socialization of confidence, where women are more conservative when updating their beliefs and ability to change [53]. Our finding could be attributed to the fact that men are more likely to avoid and delay help seeking compared to women [54], and believe they are more likely to develop disorders that lead to dementia. [55]. Our finding may also be explained by the higher prevalence rate of dementia among women in Australia [2], who perceive dementia as a priority issue in comparison to men [52]. Thus, women may attend more to campaigns and information distributed about dementia [19] surrounding risk factors, prevalence and reduction strategies.

Education was a contributor to motivational change, with individuals who had low (less than grade 6) education attainment perceiving fewer motivational cues to alter their lifestyle towards better brain health. Prior studies suggest an association between higher education, and possessing motivation, confidence and a belief that improvements in health can be achieved [20, 21]. However, our study included a large proportion of individuals who were highly-educated (45% had completed graduate or postgraduate studies) and may be a reason why this association was not replicated. Given that research into the motivation of older adults to reduce dementia risk is in its infancy, there is yet to be significant findings relating to what influences highly educated individuals to retain higher motivation levels towards health behaviours. Some researchers propose that this is the result of a flow-on effect of a well-educated population where establishing accurate health beliefs and knowledge in early education, may support initial motivations to make better lifestyle choices later. This can create better skills and greater self-advocacy with new knowledge furthering their literacy and supporting effective habit development [56, 57]. Similarly, education is tied closely to income, skills and opportunities that older adults have to lead healthy lives in their communities [58], and thus are more likely to have better environmental cues to support lifestyle change.

Our findings revealed motivation trends by health behaviours and conditions, particularly in relation to increased alcohol consumption, high cholesterol and obesity. Individuals with higher alcohol consumption perceived less benefits and social cues to action, whilst those with obesity perceived more benefits, and individuals with high cholesterol had greater perceived susceptibility. This could be due to the socialization of alcohol consumption within Australian culture [59]; prevalence of alcohol dependency and coping, especially amongst individuals with lower socioeconomic status [60]; fear of physical consequences of alcohol withdrawal [61], and exposure to alcohol-promoting advertisements which compete with, and limit the effect of public health efforts to reduce alcohol consumption [62, 63].

Thus, cues to action do not prompt a desire to change and the perceived benefits of reducing alcohol are minimal. Regarding those with high cholesterol, the condition is relatively asymptomatic yet consequences for cardiovascular health are severe [64]; health literacy on the topic is poor; patient-doctor communication about risk is unclear [65]; and monitoring whether pharmaceutical interventions or lifestyle adjustments are having an effect on cholesterol levels is difficult to observe without seeking medical assessment (e.g., blood test) [66]. Therefore perceived benefits of behaviour change are less realised, and older adults believe they are predisposed to other neurological diseases like dementia [21]. In contrast, obesity has observable and persistent detrimental impacts on physical function (e.g., falling) and wellbeing [67]. However, resources and lifestyle interventions depicting strategies to maintain a healthy weight range are readily accepted [68], effective, require minimal effort [69], and are extrinsically rewarding due to body changes [70]. Thus, older adults with obesity are more able to actively visualize the benefits of changing their behaviour, and reducing dementia is an added bonus.

### Motivation associated with dementia risk score

To date, research surrounding dementia risk is still in its infancy. Our results contribute to closing the gap and provide the initial landscape of factors influencing dementia risk. The present cohort demonstrated moderately good brain health (mean score of − 2.8) and up to 8.8% achieved the highest possible score, suggesting that these older Australians appeared to be at a lower risk of dementia as compared to other studies with a mean LIBRA score of + 1.2 [71], + 1.5 [32] and + 3.2 [28].

Understanding motivational factors that may affect high or low dementia risk has benefits in terms of developing interventions. Our findings suggest that targeting older men and people from low socioeconomic areas would lead to greater improvements in brain health than targeting other groups. Similarly, informing people about their risk to increase their perception of susceptibility and ensure that key barriers are removed will likely increase brain health. Further work is required to identify the kinds of behaviour change interventions that will best meet these needs.

Socioeconomic status has significant implications for individuals to access education and afford healthcare, with impacts on healthy longevity [72]. Consistent with previous research, our results suggest that the influence of socioeconomic status is beyond the immediate financial gains attributed to wealth, education and household income but also permeates an individual’s motivation and attitudes towards their own health. Individuals with lower socioeconomic status are more focused on the present and less so for the distant future. This is mainly due to an unliveable wage, resource scarcity, and the need to meet urgent basic requirements like food and housing. Thus, their decision-making process in areas of health shift further away from making long-term gains [73].

However, other research has highlighted that lifestyles (personal routines and behavioural patterns) have a significant mediating effect on socioeconomic status and health [74] and explains socioeconomic disparities in dementia risk [29]. Individuals who engage in more health-promoting behaviours (e.g., participate in exercise, attend social and cultural events, engage in continuous learning) have better physical and psychological health despite their socioeconomic status [74]. Indeed, enabling individuals by incorporating elements of social cohesion for engagement, provision of affordable, accessible information and tailored advice from credible sources (e.g., GPs), and constant external monitoring due to a lack of self-mobilisation have been critical to the successful implementation of grass-root lifestyle interventions in lower socioeconomic areas [75, 76].

Complementary, are whole population approaches which influence the social and environmental context of communities by reducing socioeconomic inequalities through passive engagement, tailoring and distributing resources according to level of disadvantage, and targeting deprived areas [77]. However, policy is often hesitant to implement such changes as benefits are only realized in the long-term and synergy between multiple levels of government is required [77]. Our sample were relatively healthy, had low dementia risk, were more aware of the link between lifestyle and dementia, and agreed that social influences would help to change their lifestyle and health behaviour. Thus, population health approaches combined with enabling individuals at the community level to pursue realistic healthier lifestyle choices may actualize benefits in overall health and dementia risk status.

### Limitations

Our study had both strengths and limitations. One of our biggest strengths is adopting strategies to ensure a large sample size and adequate representation of sociodemographic factors, which resulted in a good distribution of men to women and respondents from regional areas. However, we recruited a convenience sample, respondents were still from high socioeconomic backgrounds, had relatively good to moderate brain health scores and were predominantly born in English-speaking countries. Consequently, results observed may not be applicable to broader Australian society. Furthermore, as most respondents were from a high socioeconomic background and had advanced educational levels, there is potential for the study to overestimate motivational levels when generalizing findings to the Australian public.

### Implications

Previous research has surmised that by adapting strategies that address specific barriers and needs of the older population to target modifiable risk factors thoroughly and interactively in public health prevention initiatives, dementia risk can be reduced [78, 79]. Our findings provide an understanding of how motivational factors influence dementia risk. Our study suggests that improving awareness of dementia literacy and reducing lifestyle barriers may provide benefit in reducing dementia risk.

## Conclusion

Our findings provide insight into the health conditions and sociodemographic factors influencing motivation to change behaviours towards dementia risk. The weak association between perceived barriers and dementia risk suggests that motivational factors have some, but yet to be further confirmed, impact level of dementia risk. However, perceived benefits seem to be an initial driver of attitudes towards engaging in behaviours that reduce dementia risk. Investigating the impact of such lifestyle interventions and individual-level engagement should be considered to address health conditions that were found to affect motivation. Our findings also provide directions for future research to engage with other minority and disadvantaged groups (e.g., culturally and linguistically diverse older adults) and evaluate motivational, sociodemographic, culturally-specific, health and other related factors that could impact their dementia risk.

## Electronic supplementary material

Below is the link to the electronic supplementary material.

Supplementary Material 1

## Data Availability

Composite data can be made available upon reasonable request to the corresponding author (joyce.siette@westernsydney.edu.au).
