# Correction: Cross-sectional survey of attitudes and beliefs towards dementia risk reduction among Australian older adults

**DOI:** 10.1186/s12889-023-16088-7

**Published:** 2023-06-16

**Authors:** Joyce Siette, Laura Dodds, Kay Deckers, Sebastian Köhler, Christopher J. Armitage

**Affiliations:** 1 MARCS Institute for Brain, Behaviour and Development, Western Sydney University, Sydney, NSW 2145 Australia; 2 Centre for Health Systems and Safety Research, Australian Institute of Health Innovation, Macquarie University, Macquarie Park, NSW 2109 Australia; 3 Alzheimer Centrum Limburg, Department of Psychiatry and Neuropsychology, School for Mental Health and Neuroscience, Maastricht University, Maastricht, 6200 MD the Netherlands; 4 Manchester Centre for Health Psychology, University of Manchester, Manchester, M13 9PL UK; 5 Manchester University NHS Foundation Trust, Manchester Academic Health Science Centre, Manchester, M13 9PL UK; 6 NIHR Greater Manchester Patient Safety Translational Research Centre, University of Manchester, Manchester, M13 9PL UK

**Correction: BMC Public Health 23, 1021 (2023)**

**https://doi.org/10.1186/s12889-023-15843-0**

The original publication of this article contained a typo on page 6 related to the economic status of participants. The incorrect and correct information is listed in this correction article. The original article has been updated.

Incorrect

After accounting for other factors in the final linear regression model, women (ß = -0.35, *p* = 0.02), younger participants (ß = -0.72, *p* = 0.001), participants who were from a lower socioeconomic status(ß = 0.99, *p* < 0.01), and those reporting less perceived susceptibility (ß = 0.05, *p* = 0.04), more benefits (ß = 0.07, *p* = 0.03) and fewer barriers (ß = 0.10, *p* < 0.05) to adopting a healthy lifestyle had significantly lower dementia risk scores (R2 = 0.97, F(18,745) = 3.56, *p* < 0.001) (Table 4).

Correct

After accounting for other factors in the final linear regression model, women (ß = -0.35, *p* = 0.02), younger participants (ß = -0.72, *p* = 0.001), participants who were from a higher socioeconomic status(ß = 0.99, *p* < 0.01), and those reporting less perceived susceptibility (ß = 0.05, *p* = 0.04), more benefits (ß = 0.07, *p* = 0.03) and fewer barriers (ß = 0.10, *p* < 0.05) to adopting a healthy lifestyle had significantly lower dementia risk scores (R2 = 0.97, F(18,745) = 3.56, *p* < 0.001) (Table 4).
