# Supplementary material for: Cross-sectional survey of attitudes and beliefs towards dementia risk reduction among Australian older adults
Source: BMC Public Health. 2023 May 30;23:1021. doi: 10.1186/s12889-023-15843-0 (PMC10227810; doi:10.1186/s12889-023-15843-0)
Supplement: Supplementary file 1 — Supplementary Material 1 [file 12889_2023_15843_MOESM1_ESM.docx]

**Supplementary Material.**

**Supplementary Methods**

**Motivation to Change Lifestyle and Health Behaviors for Dementia Risk Reduction (MCLHB-DRR) scale**

Subscale examples

1. Consequences of the condition (perceived severity, five items; e.g., “When I think about dementia I feel nauseous”)
2. The risk of getting the condition (perceived susceptibility, four items; e.g., “My chances of developing dementia are great”),
3. Perceived barriers (four items; e.g., I am too busy to change my lifestyle and health habits”)
4. Benefits of engaging in the health-promoting behaviour (four items; e.g., I have a lot to gain by changing my lifestyle and health behaviour”)
5. General health motivation (4 items, e.g., “I often think about my health”)
6. Self-efficacy (2 items, e.g., “I am certain that I can change my lifestyle and behaviour so I can reduce the risk of developing dementia”)
7. External stimuli that may trigger an individual to take action (cue to action, 4 items; e.g., “Learning more about dementia from the media makes me think I have to change my lifestyle and behaviour”)

**Table S1. ANOVA analyses to explore differences in motivation to change lifestyle and health behaviours for dementia risk reduction by demographic variables.**

|  | **Susceptibility** | **p-value** | **Severity** | **p-value** | **Benefits** | **p-value** | **Barriers** | **p-value** | **Cues to action** | **p-value** | **General health motivation** | **p-value** | **Self-efficacy** | **p-value** |
| --- | --- | --- | --- | --- | --- | --- | --- | --- | --- | --- | --- | --- | --- | --- |
| **Gender** |  |  |  |  |  |  |  |  |  |  |  |  |  |  |
| Women | 10.9 [3.4] | 0.23 | **15.7 [3.3]** | **0.002** | 14.9 [2.5] | 0.68 | 8.3 [2.7] | 0.23 | 12.8 [2.8] | 0.05 | 15.6 [2.4] | 0.71 | 7.5 [1.3] | 0.15 |
| Men | 11.3 [3.1] |  | 14.8 [3.8] |  | 14.8 [2.7] |  | 8.1 [2.6] |  | 12.3 [3.0] |  | 15.5 [2.6] |  | 7.5 [1.6] |  |
| **Age** |  |  |  |  |  |  |  |  |  |  |  |  |  |  |
| 65-69 | 11.6 [3.3] | 0.05 | **16.0 [3.3]** | **0.01** | **15.3 [2.5]** | **<0.001** | 8.4 [2.8] | 0.21 | **13.0 [3.0]** | **0.01** | **15.9 [2.2]** | **<0.001** | **7.6 [1.4]** | **<0.001** |
| 70-79 | 11.0 [3.2] |  | 15.5 [3.3] |  | 15.2 [2.4] |  | 8.3 [2.6] |  | 12.7 [2.7] |  | 15.8 [2.4] |  | 7.7 [1.4] |  |
| 80+ | 10.8 [3.4] |  | 15.0 [3.9] |  | 14.6 [2.5] |  | 8.0 [2.6] |  | 12.4 [2.8] |  | 15.3 [2.7] |  | 7.4 [1.5] |  |
| **Country of birth** |  |  |  |  |  |  |  |  |  |  |  |  |  |  |
| English-speaking country | 11.2 [3.3] | 0.21 | 15.4 [3.5] | 0.65 | 14.8 [2.6] | 0.18 | 8.2 [2.6] | 0.51 | 12.7 [2.8] | 0.09 | **15.5 [2.4**] | **0.01** | 7.4 [1.4] | 0.38 |
| Non-English speaking country | 12.4 [1.5] |  | 17.1 [3.0] |  | 16.3 [1.8] |  | 9.8 [3.1] |  | 14.0 [3.5] |  | 17.8 [2.2] |  | 8.0 [1.5] |  |
| **Education** |  |  |  |  |  |  |  |  |  |  |  |  |  |  |
| Low | 11.4 [3.3] | 0.18 | 15.4 [3.6] | 0.11 | 15.0 [2.7] | 0.614 | 8.7 [2.6] | 0.053 | 13.1 [2.7] | **0.02** | 16.3 [2.4] | **0.01** | 7.8 [1.4] | 0.21 |
| Middle | 11.3 [3.4] |  | 15.8 [3.5] |  | 15.0 [2.6] |  | 8.5 [2.7] |  | 12.9 [2.9] |  | 15.6 [2.7] |  | 7.5 [1.5] |  |
| High | 10.9 [3.2] |  | 15.2 [3.5] |  | 14.8 [2.5] |  | 8.1 [2.6] |  | 12.4 [2.8] |  | 15.5 [2.4] |  | 7.5 [1.4] |  |
| **Socioeconomic status** |  |  |  |  |  |  |  |  |  |  |  |  |  |  |
| 1 (lowest) | 11.7 [2.0] | 0.68 | 16.4 [1.2] | 0.62 | **15.1 [2.2]** | **0.01** | **11.3 [2.4]** | **0.004** | 12.2 [1.7] | 0.44 | 15.5 [2.5] | 0.25 | 7.5 [.8] | 0.27 |
| 2 | 11.6 [3.1] |  | 15.6 [2.5] |  | 15.5 [2.4] |  | 8.1 [2.9] |  | 13.2 [2.9] |  | 14.9 [2.6] |  | 7.3 [1.3] |  |
| 3 | 11.0 [3.3] |  | 15.2 [3.5] |  | 14.8 [2.7] |  | 8.6 [2.3] |  | 12.4 [2.9] |  | 16.0 [2.3] |  | 7.6 [1.4] |  |
| 4 | 10.9 [3.2] |  | 15.0 [3.7] |  | 14.8 [2.4] |  | 8.2 [2.6] |  | 12.9 [2.5] |  | 15.7 [2.0] |  | 7.5 [1.3] |  |
| 5 (highest) | 11.4 [3.4] |  | 15.4 [3.5] |  | 15.3 [2.3] |  | 8.6 [2.4] |  | 12.6 [2.7] |  | 15.5 [2.6] |  | 7.4 [1.6] |  |
| **Locality** |  |  |  |  |  |  |  |  |  |  |  |  |  |  |
| Major city | 10.9 [3.3] | 0.06 | 15.5 [3.6] | 0.34 | 14.9 [2.6] | 0.93 | 8.3 [2.7] | 0.74 | 12.6 [2.9] | 0.95 | **15.7 [2.5]** | **0.01** | 7.5 [1.5] | 0.32 |
| Regional/Remote | 11.8 [3.1] |  | 15.1 [3.5] |  | 15.0 [2.5] |  | 8.4 [2.4] |  | 12.5 [2.8] |  | 15.5 [2.5] |  | 7.5 [1.3] |  |
| **Health condition** |  |  |  |  |  |  |  |  |  |  |  |  |  |  |
| **Heart condition** |  |  |  |  |  |  |  |  |  |  |  |  |  |  |
| Yes | 11.4 [3.1] | 0.09 | 15.3 [3.5] | 0.62 | 14.9 [2.7] | 0.77 | 8.4 [2.6] | 0.53 | 12.7 [2.8] | 0.63 | 15.6 [2.9] | 0.91 | 7.4 [1.5] | 0.27 |
| No | 10.9 [3.3] |  | 15.5 [3.5] |  | 14.9 [2.5] |  | 8.3 [2.7] |  | 12.6 [2.9] |  | 15.6 [2.3] |  | 7.5 [1.4] |  |
| **Kidney disease** |  |  |  |  |  |  |  |  |  |  |  |  |  |  |
| Yes | 11.2 [3.3] | 0.80 | 15.0 [3.9] | 0.56 | 14.6 [2.2] | 0.57 | 9.0 [2.8] | 0.18 | 12.3 [2.4] | 0.53 | 15.1 [3.3] | 0.33 | 7.4 [1.3] | 0.60 |
| No | 11.1 [3.3] |  | 15.4 [3.5] |  | 14.9 [2.6] |  | 8.3 [2.6] |  | 12.6 [2.9] |  | 15.6 [2.5] |  | 7.5 [1.4] |  |
| **Diabetes** |  |  |  |  |  |  |  |  |  |  |  |  |  |  |
| Yes | 11.2 [3.3] | 0.68 | 15.4 [3.5] | 0.94 | 15.2 [2.4] | 0.27 | 8.6 [2.5] | 0.39 | 12.9 [3.0] | 0.33 | 16.0 [2.4] | 0.14 | 7.6 [1.4] | 0.55 |
| No | 11.0 [3.3] |  | 15.4 [3.5] |  | 14.8 [2.6] |  | 8.3 [2.7] |  | 12.6 [2.8] |  | 15.6 [2.5] |  | 7.5 [1.4] |  |
| **High cholesterol** |  |  |  |  |  |  |  |  |  |  |  |  |  |  |
| Yes | **11.3 [3.1]** | **0.01** | 15.4 [3.4] | 0.78 | 15.0 [3.5] | 0.18 | 8.3 [2.6] | 0.85 | 12.8 [2.6] | 0.07 | 15.6 [2.4] | 0.70 | 7.5 [1.3] | 0.29 |
| No | 10.8 [3.4] |  | 15.4 [3.7] |  | 14.7 [2.7] |  | 8.2 [2.6] |  | 12.4 [3.0] |  | 15.6 [2.6] |  | 7.4 [1.5] |  |
| **High blood pressure** |  |  |  |  |  |  |  |  |  |  |  |  |  |  |
| Yes | 11.2 [3.1] | 0.17 | 15.6 [2.4] | 0.17 | 15.0 [2.5] | 0.12 | **8.5 [2.6]** | **0.04** | 12.7 [2.6] | 0.40 | 15.5 [2.6] | 0.60 | 7.5 [1.4] | 0.76 |
| No | 10.9 [3.4] |  | 15.2 [3.6] |  | 14.7 [2.6] |  | 8.1 [2.7] |  | 12.5 [3.0] |  | 15.6 [2.3] |  | 7.5 [1.5] |  |
| **Smoking** |  |  |  |  |  |  |  |  |  |  |  |  |  |  |
| Yes | **12.8 [3.3]** | **0.03** | 16.6 [2.4] | 0.19 | 14.6 [2.8] | 0.68 | **10.1 [3.2]** | **0.01** | 13.3 [3.6] | 0.33 | 15.4 [2.4] | 0.79 | 7.4 [1.5] | 0.71 |
| No | 11.0 [3.3] |  | 15.4 [3.5] |  | 14.9 [2.6] |  | 8.3 [2.6] |  | 12.6 [2.8] |  | 15.6 [2.5] |  | 7.5 [1.4] |  |
| **Obesity** |  |  |  |  |  |  |  |  |  |  |  |  |  |  |
| Yes | **12.8 [3.3]** | **0.03** | 16.6 [2.4] | 0.19 | 14.6 [2.8] | 0.68 | 10.1 [3.2] | 0.01 | 13.3 [3.6] | 0.33 | 15.4 [2.4] | 0.79 | 7.4 [1.5] | 0.71 |
| No | 11.0 [3.3] |  | 15.4 [3.5] |  | 14.9 [2.6] |  | 8.3 [2.6] |  | 12.6 [2.8] |  | 15.6 [2.5] |  | 7.5 [1.4] |  |
| **Depression** |  |  |  |  |  |  |  |  |  |  |  |  |  |  |
| **Yes** | 12.2 [3.4] | 0.41 | 15.3 [3.2] | 0.30 | 14.6 [3.4] | 0.26 | 9.6 [3.7] | 0.62 | 11.8 [4.4] | 0.33 | 15.5 [4.6] | 0.42 | 7.5 [2.5] | 0.20 |
| **No** | 10.3 [3.9] |  | 14.8 [4.6] |  | 14.3 [3.9] |  | 7.9 [3.1] |  | 12.0 [3.9] |  | 14.8 [4.1[ |  | 7.1 [2.1] |  |
| **Alcohol** |  |  |  |  |  |  |  |  |  |  |  |  |  |  |
| **Yes** | 10.7 [3.8] | 0.25 | **14.6 [4.5]** | **0.04** | 13.9 [3.8] | 0.11 | 7.8 [3.2] | 0.26 | **11.3 [4.0]** | **0.02** | 14.8 [4.1] | 0.16 | 7.0 [2.1] | 0.08 |
| **No** | 10.7 [4.1] |  | 14.9 [4.6] |  | 14.4 [3.8] |  | 8.0 [3.1] |  | 12.2 [3.8] |  | 14.9 [4.1] |  | 7.2 [2.1] |  |

*Values presented in means [SD], unless otherwise indicated.*

**Table S2. Summary of LIBRA scores.**

| LIBRA (mean, [SD], range)^1^ | -2.8 [2.0] (-5.9-3.9) |
| --- | --- |
| Heart disease^2^ | 208 (24.3) |
| Kidney disease | 26 (3.0) |
| Diabetes | 71 (8.3) |
| Cholesterol | 433 (50.5) |
| Smoking | 16 (1.9) |
| Obesity | 203 (23.7) |
| High blood pressure | 394 (46.0) |
| Physical inactivity | 443 (51.7) |
| Excessive alcohol consumption | 176 (20.5) |
| Poor diet | 33 (3.9) |
| Cognitive inactivity | 163 (19.0) |

*^1^ LIBRA=Lifestyle for BRAin health index ^2^ The frequency and proportion refers to the presence of individual risk factors. SD=Standard deviation.*

**Table S3. ANOVA analyses of dementia risk score by demographic and health variables.**

|  | LIBRA Index Mean (SD) | F | p-value |
| --- | --- | --- | --- |
| Gender |  |  |  |
| Women | -2.9 (1.9) | 4.25 | **0.04** |
| Men | -2.6 (2.0) |  |  |
| Age |  |  |  |
| 65-69 | -2.9 (1.8) | 1.74 | 0.16 |
| 70-79 | -2.8 (2.0) |  |  |
| 80+ | -2.5 (2.1) |  |  |
| Country of birth |  |  |  |
| English-speaking country | -2.8 (1.9) | 0.32 | 0.57 |
| Non-English speaking country | -2.9 (2.1) |  |  |
| Locality |  |  |  |
| Major city | -2.9 (2.0) | 3.56 | 0.06 |
| Regional/Remote | -2.5 (1.8) |  |  |
| Education |  |  |  |
| Low | -2.8 (2.0) | 1.29 | 0.277 |
| Middle | -2.6 (1.9) |  |  |
| High | -2.9 (1.9) |  |  |
| Socioeconomic status |  |  |  |
| 1 (lowest) | -1.9 (2.2) | 4.75 | **<0.001** |
| 2 | -2.6 (2.0) |  |  |
| 3 | -2.5 (1.9) |  |  |
| 4 | -2.9 (1.9) |  |  |
| 5 (highest) | -3.0 (1.9) |  |  |
| Locality |  |  |  |
| Metropolitan | -2.9 (2.0) | 3.56 | 0.06 |
| Regional/Remote | -2.5 (1.8) |  |  |

*Values presented in means [SD], unless otherwise indicated. LIBRA=Lifestyle for BRAin health index*
